# Cochrane diagnostic test accuracy reviews

**DOI:** 10.1186/2046-4053-2-82

**Published:** 2013-10-07

**Authors:** Mariska MG Leeflang, Jonathan J Deeks, Yemisi Takwoingi, Petra Macaskill

**Affiliations:** 1 Department of Clinical Epidemiology, Biostatistics and Bioinformatics, Academic Medical Center, Meibergdreef 9, 1105 AZ, Amsterdam, The Netherlands; 2 Biostatistics, Evidence Synthesis and Test Evaluation Research Group, School of Health and Populations Sciences, Public Health Building, University of Birmingham, Edgbaston, Birmingham B15 2TT, UK; 3 Screening and Test Evaluation Program, School of Public Health, University of Sydney, Sydney NSW 2006, Australia

## Abstract

In 1996, shortly after the founding of The Cochrane Collaboration, leading figures in test evaluation research established a Methods Group to focus on the relatively new and rapidly evolving methods for the systematic review of studies of diagnostic tests. Seven years later, the Collaboration decided it was time to develop a publication format and methodology for Diagnostic Test Accuracy (DTA) reviews, as well as the software needed to implement these reviews in The Cochrane Library. A meeting hosted by the German Cochrane Centre in 2004 brought together key methodologists in the area, many of whom became closely involved in the subsequent development of the methodological framework for DTA reviews. DTA reviews first appeared in The Cochrane Library in 2008 and are now an integral part of the work of the Collaboration.

## Background

Finding good evidence regarding the performance of diagnostic tests and interpreting its value for practice is more challenging and less straightforward than for interventions. Most diagnostic studies focus on diagnostic test accuracy, which expresses a test’s ability to discriminate between people with the target condition and those without it [see Additional file 1]. However, estimates of test accuracy often vary markedly between studies. Such heterogeneity may reflect differences between studies in the criterion used to define test positivity, study design and patient characteristics as well as the place of the test in the diagnostic pathway [1-3]. Furthermore, a highly accurate test does not necessarily improve a patient’s outcome [4]. Systematic reviews of diagnostic test accuracy summarize the evidence about test accuracy. Ideally, they also investigate why the results may vary among studies, compare the performance of alternative tests, and help the reader to put the evidence in a clinical context [5,6].

In the early 1990s, several researchers led by Les Irwig and Paul Glasziou were working on methods for the systematic review of diagnostic test accuracy and identified The Cochrane Collaboration as an obvious place where health professionals looking for evidence about diagnostic tests should be able to go. After an initial meeting at the 2^nd^ Cochrane Colloquium in Hamilton, Ontario on 2 October 1994, the Cochrane Screening and Diagnostic Test Methods Group was founded and formally registered in the Collaboration in 1996. It initially focused on identifying a common method for preparing diagnostic test accuracy reviews.

One of their goals was to include diagnostic test accuracy (DTA) reviews in The Cochrane Library. However, largely because of the limited resources available, the Steering Group of The Cochrane Collaboration decided that, in 1996, the Collaboration was not ready to include such a methodologically challenging review type. Seven years later, in 2003, Jon Deeks and Constantine Gatsonis persuaded the Collaboration to revisit the question of inclusion of DTA reviews. The Cochrane Collaboration was then ten years old and had proven its value for decisions about interventions, and important advances had been made on the methodology for diagnostic test accuracy reviews. The Collaboration decided that the time was right to plan for the inclusion of systematic reviews of diagnostic test accuracy studies in The Cochrane Library. A Cochrane Diagnostic Reviews Working Group, led by Jon Deeks, Constantine Gatsonis and Patrick Bossuyt with members of the Methods Group, software experts, editors of Cochrane Review Groups and interested authors was established to plan and undertake the work required for the Collaboration to deliver on these reviews [see Additional file 2].

The first step involved achieving consensus on a core method. The following year, the proposers of the Bayes’ Library (led by Matthias Egger and Daniel Pewsner), members of the Cochrane Screening and Diagnostic Test Methods Group, and other international experts met together in Freiburg, Germany, to discuss and agree on appropriate methods for each step in a meta-analysis of diagnostic test accuracy, including graphical displays. The Bayes’ Library proposal was radically different in that it considered producing a database of meta-analytical estimates of likelihood ratios and pre-test probabilities, which could be used for probability revision in Bayesian diagnostic thinking. After debate, consensus was reached on following a more standard methodology that utilised sensitivity and specificity estimates. Following the meeting, members of the Cochrane Screening and Diagnostic Test Methods Group assisted Collaboration’s Information Management Team with the development of a version of the Collaboration’s Review Manager software including functions necessary for DTA reviews and worked with the Collaboration’s publisher to develop a publication format. Unlike the software for intervention reviews, which includes the ability to calculate and display the results of meta-analyses of the included studies, an approach was taken for linking the Collaboration’s software with commercial statistical software packages that contained the functionality necessary to fit the complex hierarchical statistical models for meta-analysis.

The Cochrane Library was ready to register titles for diagnostic test accuracy reviews in October 2007, with the publication of the first Cochrane diagnostic test accuracy review in October 2008 [7]. During this period, members of the Cochrane Screening and Diagnostic Test Methods Group worked not only on the development of the above mentioned methods, but also on the development of pilot reviews and guidance in the form of a Handbook. Support Units were established in the United Kingdom and The Netherlands to assist the Cochrane Review Groups with publication preparation and processes surrounding these reviews; a website was launched, training workshops were provided and a separate Editorial Team was established to oversee DTA reviews [8].

In the following sections, we highlight some of the methodological developments in diagnostic systematic reviews that took place from the early 1990s until now, against the background of the history outlined above. Current challenges and possible solutions for them are discussed, and we conclude with an overview of the current status of these reviews within The Cochrane Collaboration.

### Early methodology

The first meta-analyses of diagnostic test accuracy were published in the late 1980s and early 1990s and largely followed the approaches used for intervention meta-analyses: retrieval and selection of studies, assessing their quality, summarizing their results in a meta-analysis, investigating heterogeneity and drawing conclusions for example, [9,10]. However, meta-analysis of diagnostic test accuracy was intrinsically more complex because test accuracy measures usually come in pairs: sensitivity and specificity; positive and negative predictive values; and positive and negative likelihood ratios. A key consideration is that accuracy measures depend on the threshold that is used to define a positive test result. Sensitivity and specificity, which are commonly reported, vary in opposite directions as the threshold changes. An early regression based method that did take this into account was not straightforward to fit [10]. Another approach used the area under the receiver operating characteristic (ROC) curve to provide a single summary measure of accuracy per study, thus losing information about threshold effects [11]. A major breakthrough in the meta-analysis of diagnostic test accuracy was the publication of the statistical method developed by Moses, Littenberg and colleagues, which was straightforward to implement and also took the threshold effect into account [12,13]. This method was widely adopted in subsequent reviews.

The complexity of DTA reviews is not restricted to statistical methods. Even formulating the review question may not be straightforward because the accuracy of a test can vary in different situations. For instance, study design may affect estimated accuracy, and there is no ‘best’ design analogous to the use of the randomized trial to compare interventions. Furthermore, there is no standard terminology to describe the variety of study designs used to assess accuracy. Consequently, it is more difficult to retrieve relevant studies from electronic databases and the selection process is more complex. Interpretation of summary estimates from a DTA review also requires careful consideration because a highly accurate test in itself will not improve the patient’s outcome. It is the management of the patient and decisions made after the test is administered that directly influence the patient’s wellbeing. These more epidemiological issues and considerations for the meta-analysis of test accuracy studies were published in parallel with the statistical developments [5,14]. After almost 20 years, these guidelines [5] are still very relevant and current.

### Recent developments

At the time that the Cochrane Collaboration Steering Group decided that it would consider diagnostic test accuracy reviews, it appeared that the methods for these reviews were well defined [15,16] and all that remained was to reach consensus about which methods to adopt. However, as the discussions progressed, limitations of existing commonly used approaches became clear, and ideas for alternative methods and further developments were generated. These are outlined below.

#### Question formulation and the interpretation of results

There was an increasing awareness that because tests are used in a range of contexts, their value very much depends on their place and role in clinical practice [17]. This also affects the interpretation and applicability of the findings: Do the findings hold for any situations, or do different situations cause the test to behave differently? For example, questionnaires to determine whether elderly patients are developing dementia may be of value in general practice. However, when such a questionnaire is used in a mental health clinic where patients have many multiple symptoms in common, the questionnaire is no longer able to distinguish between someone with general mental impairment and someone with dementia.

Even if such a questionnaire could distinguish very well between people with general cognitive impairment and someone with dementia, its value may still depend on other factors such as whether the knowledge that someone has dementia rather than general cognitive impairment will affect their outcomes and quality of life. The potential consequences of a positive or negative test result should be taken into account when interpreting the results of a DTA review. If knowledge of the test result does not affect further management, the value of testing at that point may be very limited.

When formulating the review question, one should also realize that diagnostic tests are not used in isolation and that alternatives should be considered as well. Therefore, Cochrane DTA reviews have also turned their focus on the importance of comparative accuracy, because choosing a test requires robust information about the value it adds compared to existing alternatives.

#### Search and selection

Studies of the relative effects of different intervention are relatively easy to find by searching for randomized trials. Searching for studies of diagnostic test accuracy is far more difficult because the study designs vary and there is no one term that can be used to filter all diagnostic studies. Multiple combinations of methodological terms have been tried, resulting in the development of so called ‘methodological search filters’. However, it has become clear that searching for diagnostic accuracy studies involves more than filtering studies for their use of diagnosis-related terms [18,19]. As a result, review authors are often forced to screen thousands of retrieved article titles in order to find a relatively small number of potentially relevant studies.

#### Quality assessment

The first published empirical investigation of the effect of a range of potential biases on diagnostic accuracy outcomes was published in 2002 [20]. An overview of all potential sources of bias and variation was published two years later and formed the basis of a Quality Assessment for Diagnostic Accuracy Studies (QUADAS) tool [21,22]. This tool consisted of 14 items and has been widely used by authors of diagnostic test accuracy reviews. A modified form of QUADAS became the recommended quality assessment tool for Cochrane diagnostic accuracy reviews [23].

As the tool became more widely used, it became apparent that it had some drawbacks such as not distinguishing adequately between true biases and reporting biases, and also not distinguishing between risk of bias and issues of applicability or representativeness. In response to these limitations, an updated version of the tool was developed and published in 2011 [24]. This version, which is now used for Cochrane DTA reviews, allows the assessment of both risk of bias and concerns regarding applicability in an explicit and transparent way.

#### Meta-analysis

As outlined above, the statistical approach developed by Moses and Littenberg was widely adopted as it was straightforward to apply and understand. Alternative, but substantially more complex statistical approaches were published in the mid 1990s, providing a framework for more rigorous methods taking proper account of within study variability in sensitivity and specificity, and unexplained heterogeneity in test accuracy between studies. [25,26]. These more rigorous methods are the basis for the hierarchical models that are recommended for Cochrane DTA reviews and that are increasingly used in preference to the original Moses and Littenberg method.

**Figure 1:**
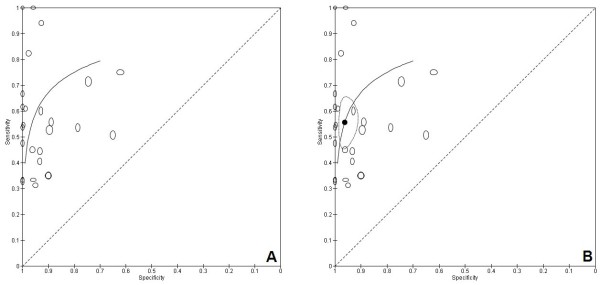
**Summary receiver-operating characteristic (ROC) plots showing test accuracy of cytology for detecting primary bladder cancer **[32]**. A)** The summary ROC curve, representing the underlying relationship between sensitivity and specificity for the test across varying thresholds. **B)** The summary sensitivity and specificity and a 95% confidence region around it. The smaller oval shaped symbols in both graphs show the individual study results, with the height of the symbol representing the number of diseased individuals and the width of the ovals representing the number of non-diseased individuals.

Both of these hierarchical models use an estimate of test sensitivity and specificity for each study. The first model, commonly referred to as the Rutter and Gatsonis Hierarchical Summary ROC (HSROC) model, focuses on the estimation of a summary ROC curve that allows for threshold effects (Figure 1A) [27]. A modification of this approach was identified to fit this model in SAS software, which has facilitated its adoption [28]. A second model, commonly referred to as the bivariate model, performs a joint meta-analysis of logit transformed sensitivity and specificity, allowing for correlation between them across studies, with the aim of obtaining a summary estimate for both sensitivity and specificity (Figure 1B) [29]. Further work on these models has demonstrated that they are mathematically equivalent, but the different parameterisations affect the interpretation of covariates included in the models [30,31].

The Rutter and Gatsonis (HSROC) model assumes that each test is subject to a threshold effect, either explicitly by applying a different cut-point in the case of continuous test results, or implicitly as occurs in imaging studies. Under the HSROC model, threshold effects between studies are accounted for by a proxy measure for threshold that is based on an underlying test positivity rate in each study. If thresholds vary between studies, estimating one overall summary pair of sensitivity and specificity is not appropriate or readily interpretable because the sensitivity and specificity will vary by threshold. The bivariate model adopted by Reitsma and colleagues focuses on the estimation of a summary pair of sensitivity and specificity on the basis that clinicians require this information to assess the consequences of decisions made after a test result is known. Clearly, this approach requires that the study specific estimates of sensitivity and specificity for a test are obtained using a common criterion (threshold) for test positivity for the summary estimates to have a clear interpretation. Because of these considerations, review authors are advised to think carefully about the questions they aim to address in their review and the type of test they are analyzing to guide their choice of model [33].

### Future developments

With most of the basic methods now developed and available as guidance for review authors [6,8], it is time to consider future directions. Some ongoing developments may make the process of preparing a systematic review of diagnostic test accuracy easier, but other developments may lead to greater complexity.

#### Search and selection

Developments in text mining and machine learning techniques may make the search and selection of studies an easier task. These techniques may help in developing search strategies, but their biggest advantage will probably be in the stages of study selection The software can be trained to recognize relevant studies from irrelevant studies, allowing automatic filtering out of the clearly non-relevant studies at the first selection stage. The techniques may also be used in place of a second or third reviewer, being more objective and perhaps also more consistent than a human reviewer. This could facilitate the handling of disagreements in the selection stage.

#### Publication bias

In diagnostic research, not much is known about the ‘drivers’ behind publication bias. A diagnostic accuracy study usually does not test a hypothesis and so there is no *P* value for authors and publishers to influence decisions about publication that are based on the statistical significance of the results. Investigating what drives the publication of a diagnostic study is difficult because no formal registration of these studies exists, and because these studies may also be done on ad-hoc basis using pre-existing data or samples. In the light of the current developments with regard to the ensuring publication of each trial ever done (see http://www.alltrials.net), it would be good to set similar standards for accuracy studies. Until then, we should urge review authors to put extra effort into finding unpublished, as well as published diagnostic test accuracy studies. This will also help to inform factors associated with non-publication, thereby informing the further development of approaches for assessing potential publication bias [34,35].

#### Meta-analysis

In terms of statistical methods, future developments are likely to reflect the increasing interest in comparative accuracy of tests. Alternative tests are generally available; hence, it is appropriate to evaluate the accuracy of a test not in isolation, but relative to relevant alternative tests. Unfortunately, studies that directly compare tests are not common and meta-analyses to compare tests must often rely on a set of studies that evaluated one of the tests (test A) and a different set of studies that have evaluated the alternative test (test B). This indirect approach would not be acceptable in a systematic review to compare the effectiveness of two interventions, but is common practice when comparing tests because of the limitations of available data. Nevertheless, developments in the area of indirect comparisons and multiple treatment comparison meta-analyses for intervention studies may help to guide future methodological developments for DTA comparative meta-analyses [36]. At present, the routinely used models for DTA meta-analysis utilise data on a single sensitivity and specificity pair for each study. Hence, current models do not fully utilise all of the available data. Some progress has been made in this area [37], but more general and robust methods are required.

#### Interpretation and summary of findings

A major focus of DTA reviews is to obtain summary estimates of test accuracy. However, knowing that a test has a high sensitivity for instance does not tell us whether the test will have much impact on the patient, nor does it tell us that using this test in practice will be beneficial for the patient, or cost-effective. Improved accuracy is not even necessary for patient benefit to occur because new tests may improve outcomes if they can be used on a wider patient group, are less invasive, or allow time-critical effective therapy to be given earlier [38]. Although a GRADE approach for diagnostic tests has now been developed, providing guidance on how to translate accuracy data into a recommendation involving patient important outcomes requires much more consideration [39].

## Conclusions

Preparing a diagnostic test accuracy review is likely to be very time consuming and challenging. The challenges start at the point of question formulation. Most chapters of the Cochrane Handbook for Diagnostic Test Accuracy Reviews have been published and software is available to facilitate the review process and meta-analysis. In April 2013, the titles for around Cochrane DTA reviews have been registered. With 13 published reviews and 61 published protocols in Issue 4, 2013 of The Cochrane Library, the DTA reviews are now an established part of the Library and may serve as an example for the inclusion of future new review types.

## Abbreviations

DTA: Diagnostic test accuracy; HSROC: Rutter and Gatsonis Hierarchical Summary ROC; QUADAS: Quality Assessment for Diagnostic Accuracy Studies; ROC: Receiver operating characteristic; GRADE: Grading of Recommendations Assessment, Development and Evaluation.

## Competing interests

ML, YT and PM are co-convenors of the Cochrane Screening and Diagnostic Test Methods Group and Editors of the Cochrane Collaboration’s Diagnostic Test Accuracy Editorial Team. JJD is editor of the Cochrane Handbook for Diagnostic Test Accuracy Reviews and Executive Editor of the Cochrane Diagnostic Test Accuracy Editorial Team.

## Authors’ contributions

ML drafted the manuscript and appendix and collected historical data and names. JJD and PM collected historical data. YT drafted the glossary. All authors edited the manuscript. All authors read and approved the final manuscript.

## Supplementary Material

Additional file 1 **Glossary.** This Glossary contains the definitions for some of the technical terms mentioned in the main text. Click here for file

Additional file 2 **Appendix.** Contributors to the Diagnostic Test Accuracy Working Group. Click here for file
