# Supplementary material for: Cochrane diagnostic test accuracy reviews
Source: Syst Rev. 2013 Oct 7;2:82. doi: 10.1186/2046-4053-2-82 (PMC3851548; doi:10.1186/2046-4053-2-82)
Supplement: Additional file 1 — Glossary. This Glossary contains the definitions for some of the technical terms mentioned in the main text. [file 2046-4053-2-82-S1.doc]

*Convenors of the Screening and Diagnostic Test Methods Group include:*

Les Irwig (1996 – 1998)

Paul Glasziou (1996 – 1998)

Constantine Gatsonis (1998 – 2011)

Frank Buntinx (1999 – 2003)

Petra Macaskill (2004 – present)

Roger Harbord (2006 – 2012)

Mariska Leeflang (2009 – present)

Yemisi Takwoingi (2012 – present)

*Leaders of the Diagnostic Test Accuracy Working Group include* (in alphabetical order)*:*

Patrick Bossuyt

Jonathan Deeks

Constantine Gatsonis

*Other contributors to the Cochrane Diagnostic Test Accuracy Working Group include* (in alphabetical order):

Bert Aertgeerts, Doug Altman, Gerd Antes, Lucas Bachmann, Heiner Buchner, Peter Bunting, Jonathan Craig, Roberto D’Amico, Clare Davenport, Jenny Doust, Matthias Egger, Anne Eisinga, Graziella Fillipini, Yngve Falck-Ytter, Simon Gilbody, Afina Glas, Paul Good, Fritz Grossenbacher, Jorgen Hilden, Lotty Hooft, Andrea Horvath, Chris Hyde, Monica Kjeldstrøm, Susan Mallett, Ruth Mitchell, Tess Moore, Rasmus Moustgaard, Wytze Oosterhuis, Madhukar Pai, Prashni Paliwal, Daniel Pewsner, Hans Reitsma, Jacob Riis, Ingrid Riphagen, Anne Rutjes, Mia Schmidt-Hansen, Rob Scholten, Nynke Smidt, Karen Steingart, Jonathan Sterne, Gautham Suresh, Arianne Verhagen, Riekie de Vet, Gianni Virgilli, Vasivy Vlassov, Tanya Walsh, Joseph Watine, Danielle van der Windt, Penny Whiting, Susie Wisniewski.
