# Supplementary material for: Cochrane diagnostic test accuracy reviews
Source: Syst Rev. 2013 Oct 7;2:82. doi: 10.1186/2046-4053-2-82 (PMC3851548; doi:10.1186/2046-4053-2-82)
Supplement: Additional file 2 — Appendix. Contributors to the Diagnostic Test Accuracy Working Group. [file 2046-4053-2-82-S2.docx]

**Glossary**

***Negative likelihood ratio:*** ratio of the proportion that test negative amongst those that have the target condition compared to the proportion that test negative amongst those who do not have the target condition.

***Negative predictive value:*** proportion that do not have the target condition amongst those that test negative.

***Positive likelihood ratio:*** ratio of the proportion that test positive amongst those that have the target condition compared to the proportion that test positive amongst those who do not have the target condition.

***Positive predictive value:*** proportion that have the target condition amongst those that test positive.

***Pre-test probability:*** proportion with the target condition amongst the group suspected of having the condition.

***Receiver characteristic operating (ROC) curve:*** the sensitivity and specificity of a test vary depending on the threshold value chosen. The ROC curve describes the trade-off between sensitivity and specificity as the threshold changes.

***Sensitivity:*** proportion that test positive amongst those having the target condition.

***Specificity:*** proportion that test negative amongst those without the target condition.

***Threshold:*** a value above or below which a test result is considered positive
